# Supplementary material for: Developmental Differences between Anthers of Diploid and Autotetraploid Rice at Meiosis
Source: Plants (Basel). 2022 Jun 22;11(13):1647. doi: 10.3390/plants11131647 (PMC9268837; doi:10.3390/plants11131647)
Supplement: Supplementary file 1 [file plants-11-01647-s001.zip › plants-1765681-supplementary.pdf]

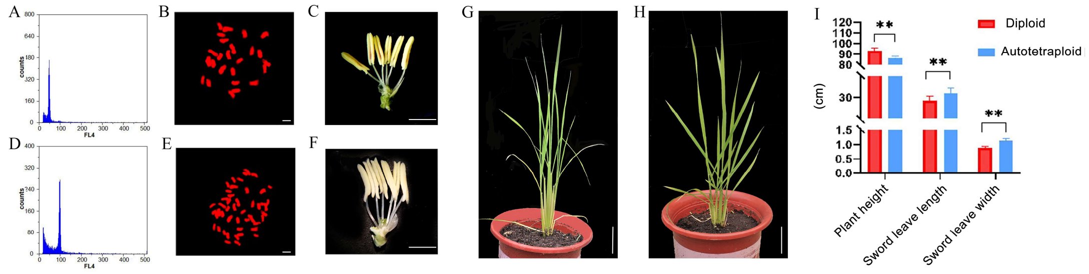

Figure S1. Ploidy detection and phenotypes of diploid rice and autotetraploid rice. A/B/C: Diploid; D/E/F: Autotetraploid. A/D: Flow cytometry, judge the ploidy by the emitted fluorescence intensity X-Mean; B/E: Chromosome count in metaphase of mitosis; C/F: Anther phenotype; G: Diploid rice; H: Autotetraploid rice; I: Comparison of plant height, sword leaf length, and sword leaf width, n=10, P<0.01. Bar = 10 μm (b, e), 1 cm (c, f), 10 cm (g, h).

Table S1. Statistics of anther size of diploid and autotetraploid rice in different.

	Before meiosis	Meiosis	Microspore stage
Diploid	0.35-0.45	0.50-1.15	1.25-1.85
Autotetraploid	0.45-0.60	0.65-1.45	1.50-2.20

Table S2. Primer information sequence.

Name	Sequence（5′ to3′）
Reference gene-F	ATCGCCCTGGACTATGAC
Reference gene-R	GAAACGCTCAGCACCAAT
BGIOSGA007251-F	GGTCAACGACGAGCCTGTTA
BGIOSGA007251-R	GTGACGAAGTACAGGGTGGG
BGIOSGA008908-F	CGCTGACAACGAGGCCATAA
BGIOSGA008908-R	CTCTGCTTCAGATGGGCGAA
BGIOSGA002684-F	TCAAGGTGCCGTCAAGGTTT
BGIOSGA002684-R	CCACCCTCTAATCCTGTCGC
BGIOSGA035762-F	CTCATCGGGATGCGCTACAT
BGIOSGA035762-R	GGCGACCCTGTATGGATCTG
BGIOSGA017916-F	AGATCAGCATCTACGACCCG
BGIOSGA017916-R	AGATCAGCATCTACGACCCG
BGIOSGA004771-F	GCAGAGGAGCOCATATCACC
BGIOSGA004771-R	CAAGTCAGCAAACCGCAACA
BGIOSGA017346-F	CACTCGGCCAAAAAGACAGC
BGIOSGA017346-R	GTCCTTGGTGCAGTTCAACG
BGIOSGA021194-F	GGCTGTCATCCATTTTGCCG
BGIOSGA021194-R	TTGTTCTGCCGTAGGGGTTC
BGIOSGA027368-F	GGCACTGCAAGAAATTGGCA
BGIOSGA027368-R	ACTCTAGCCCCCTGGTAAGG
